# Isolation, Characterization, and Complete Genome Sequence of a *Bradyrhizobium* Strain Lb8 From Nodules of Peanut Utilizing Crack Entry Infection

**DOI:** 10.3389/fmicb.2020.00093

**Published:** 2020-02-07

**Authors:** Dev Paudel, Fengxia Liu, Liping Wang, Matthew Crook, Stephanie Maya, Ze Peng, Karen Kelley, Jean-Michel Ané, Jianping Wang

**Affiliations:** ^1^Agronomy Department, University of Florida, Gainesville, FL, United States; ^2^Department of Microbiology, Weber State University, Ogden, UT, United States; ^3^Interdisciplinary Center for Biotechnology Research, University of Florida, Gainesville, FL, United States; ^4^Departments of Bacteriology and Agronomy, University of Wisconsin–Madison, Madison, WI, United States; ^5^Plant Molecular and Cellular Biology Program, Genetic Institute, University of Florida, Gainesville, FL, United States

**Keywords:** nodulation, peanut, *Bradyrhizobium*, genome, crack-entry

## Abstract

In many legumes, the colonization of roots by rhizobia is via “root hair entry” and its molecular mechanisms have been extensively studied. However, the nodulation of peanuts (*Arachis hypogaea* L.) by *Bradyrhizobium* strains requires an intercellular colonization process called “crack entry,” which is understudied. To understand the intercellular crack entry process, it is critical to develop the tools and resources related to the rhizobium in addition to focus on investigating the mechanisms of the plant host. In this study, we isolated a *Bradyrhizobium* sp. strain, Lb8 from peanut root nodules and sequenced it using PacBio long reads. The complete genome sequence was a circular chromosome of 8,718,147 base-pair (bp) with an average GC content of 63.14%. No plasmid sequence was detected in the sequenced DNA sample. A total of 8,433 potential protein-encoding genes, one rRNA cluster, and 51 tRNA genes were annotated. Fifty-eight percent of the predicted genes showed similarity to genes of known functions and were classified into 27 subsystems representing various biological processes. The genome shared 92% of the gene families with *B. diazoefficens* USDA 110^T^. A presumptive symbiosis island of 778 Kb was detected, which included two clusters of *nif* and *nod* genes. A total of 711 putative protein-encoding genes were in this region, among which 455 genes have potential functions related to symbiotic nitrogen fixation and DNA transmission. Of 21 genes annotated as transposase, 16 were located in the symbiosis island. Lb8 possessed both Type III and Type IV protein secretion systems, and our work elucidated the association of flagellar Type III secretion systems in bradyrhizobia. These observations suggested that complex rearrangement, such as horizontal transfer and insertion of different DNA elements, might be responsible for the plasticity of the *Bradyrhizobium* genome.

## Introduction

Rhizobia are soil-borne bacteria that colonize plant tissues inter- or intra-cellularly and trigger the development of new organs called nodules, which generally happens on legume roots. Inside of these nodules, the rhizobia differentiate into bacteroids and convert N_2_ gas into ammonium (Oldroyd and Downie, 2008; Kuever et al., 2015; Ormeño-Orrillo et al., 2015; Peix et al., 2015; Mus et al., 2016). This legume–rhizobium symbiosis is fundamental to sustainable agriculture as this relationship can alleviate the need to provide synthetic nitrogen fertilizers in agricultural systems (Howieson and Dilworth, 2016). In most legume–rhizobium associations, flavonoid molecules in the rhizosphere, released by plant roots, serve as signals to activate bacterial transcriptional regulator protein, NodD. The *nodD* gene controls the expression of *nod* common and specificity genes (Dénarié et al., 1996). The products of *nod* genes are involved in the synthesis and secretion of lipo-chitooligosaccharides (LCOs), called Nod factors, which are the primary determinant of specificity between rhizobia and their legume hosts (Downie and Walker, 1999; Oldroyd, 2013). Nod factor recognition at the root surface leads to the initiation of a nodule primordium in the root cortex. However, in some associations, Nod factors are not required, and the rhizobia use other strategies, such as the use of effectors (Masson-Boivin and Sachs, 2018). Nevertheless, in all associations, the rhizobia go through the epidermis and the cortex to gain access to developing nodule primordia, where the bacteria are released into cortical cells (Oldroyd et al., 2011). Rhizobial infection in the majority of legume species, such as soybean (*Glycine max*), *Medicago truncatula*, and *Lotus japonicus*, follows a “root hair entry” pathway, which involves penetrating via intracellular infection threads (Bonaldi et al., 2011; Oldroyd et al., 2011). However, intracellular infection threads are not found in the roots of legumes such as peanut (*Arachis hypogaea* L.) and *Aeschynomene*, where infection occurs intercellularly via “crack entry.” In peanut, the rhizobia enter the root, where root hairs emerge and occupy the space between the root hair wall and adjoining epidermal and cortical cells. They colonize the intercellular spaces and invade sub-epidermal cortical cells via an endocytosis-like process (Okubo et al., 2012a). The invaded cells divide and are incorporated in the nodule tissue. Adjacent cells separate at the middle lamellae, and this space is filled with bacteria, thus distributing the bacteria intercellularly (Chandler, 1978). This intercellular infection is considered an evolutionarily ancient invasion mechanism (Groth et al., 2010; Madsen et al., 2010), which might be a key to facilitate transforming non-legumes for biological nitrogen fixation via symbiosis (Charpentier and Oldroyd, 2010).

Rhizobia are known to occur in eight bacterial families: *Rhizobiaceae, Brucellaceae, Phyllobacteriaceae, Xanthobacteraceae, Bradyrhizobiaceae, Hyphomicrobiaceae, Methylobacteriaceae*, and *Burkholderiaceae* (de Lajudie et al., 2019). Among these families, *Bradyrhizobiaceae* contains the genus *Bradyrhizobium*, a slow-growing (doubling time >8 h), non-acid-producing root nodule bacterium of leguminous plants (Jordan, 1982). Bradyrhizobia are described as gram-negative, aerobic, non-spore forming, short rod-shaped (0.5–0.9 μm by 1.2–3.0 μm), and mobile by one polar or subpolar flagellum (Jordan, 1982). Currently, a total of 41 species have been assigned to the genus *Bradyrhizobium* (Parte, 2014). *Bradyrhizobium diazoefficiens*, recently reclassified from *B. japonicum* (Delamuta et al., 2013) forms symbiotic, nitrogen-fixing relationships with soybean plants and has been used in agriculture for decades (Stacey, 1995; Kaneko et al., 2002). Considering its essential role in nitrogen fixation, the complete genome of *B. diazoefficiens* USDA 110^T^, has been sequenced (Kaneko et al., 2002). Furthermore, according to the National Center for Biotechnology Information Database, 37 *Bradyrhizobium* strains isolated from different species, including soybean, *Aeschynomene*, and wheat have been sequenced. In general, clustering of nodulation and nitrogen fixation genes in plasmids or symbiotic islands is common in rhizobial genomes that colonize roots by “root hair entry” (Giraud et al., 2007). Some rhizobia of the genus *Bradyrhizobium* efficiently nodulate peanuts, the second most important legume crop in the world after soybeans (Urtz and Elkan, 1996). Even though the peanut–rhizobium symbiosis is essential and supplies 55% of nitrogen needs for optimal peanut growth (Hardarson, 1993), there is a shortage of genomic information on peanut rhizobia.

Unraveling the genomes of peanut rhizobia is particularly critical because the infection mechanism that takes place during peanut nodulation is proposed to be an ancestral state (Sprent, 2007). This extracellular mode of colonization could be a more plausible target to engineer entry of rhizobia into non-legumes than the more advanced root-hair entry (Charpentier and Oldroyd, 2010). While molecular mechanisms in intracellular root-hair entry species like *M. truncatula* and *L. japonicus* have been under extensive investigation, many questions regarding the symbiotic pathway for evolutionarily ancient extracellular pathway remain unaddressed. To further study the extracellular infection path, it is critical to not just investigate the host mechanisms in controlling infection, but also to develop the tools and resources related to rhizobium. Therefore, the objective of this study was to increase the knowledge on native bradyrhizobia that nodulate peanut by studying the genome of strain Lb8 and its phylogenetic characterization. We utilized long reads from single-molecule real-time (SMRT) sequencing (Pacific Biosciences) to assemble the complete genome of *Bradyrhizobium* sp. Lb8. Here, we provide an analysis of the first complete genome sequence of an isolated strain of *Bradyrhizobium* that efficiently fixes nitrogen in peanuts.

## Results

### Isolation of the Rhizobia Strains From Peanut Nodules

Out of 370 colony PCRs, 359 colonies showed PCR products, without a difference in amplicon size. Five PCR products that were randomly selected for sequencing revealed that the five isolates belonged to the genus *Bradyrhizobium*, with two unique 16S rRNA sequences. Strains L1, L3, and L4 had identical 16S rRNA sequences, while strains Lb8 and Ls1 had identical 16S rRNA sequences.

### Characterization of the Isolated Strains

These five *Bradyrhizobium* strains showed three different growth curves (Supplementary Figure 1). Lb8 grew the fastest in the first 8 days followed by L1, L3, and L4. The growth rate of L1, L3, and L4 continued after day 8 and surpassed Lb8 on day 9. Nodules were seen on the roots of all peanut plants inoculated with the five strains separately, at 13 days after inoculation (DAI) (Supplementary Figure 2A). Nodules were harvested at 22 DAI and immediately cut in half. The inside of the nodules was dark pink (Supplementary Figure 2B), indicating that all the five rhizobial strains isolated from peanut were able to infect peanuts to form functional nodules.

**Figure 1:**
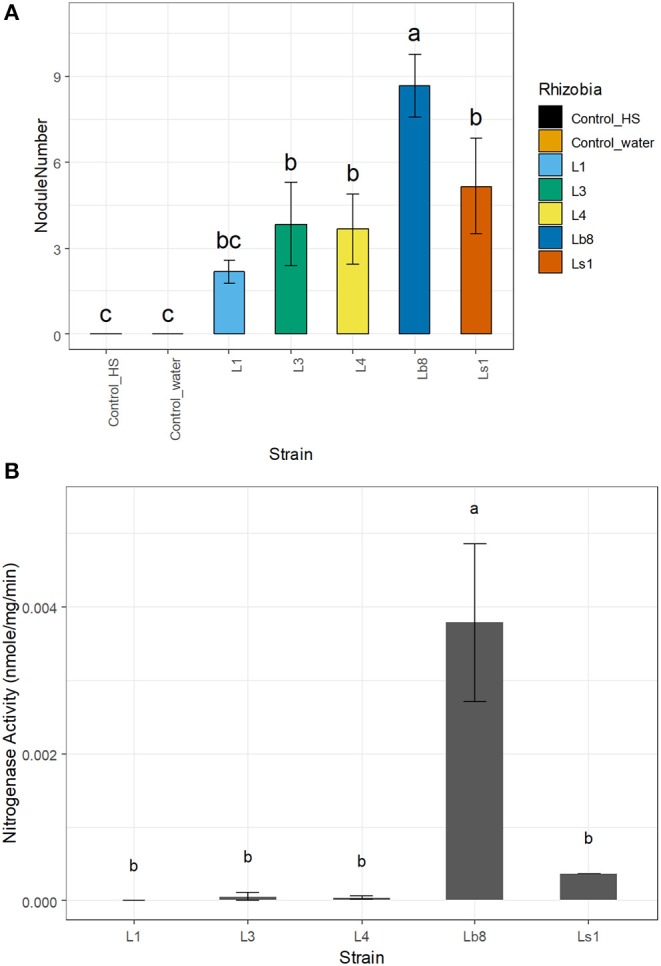
Bar plots showing the number of nodules per plant **(A)** and nitrogenase activity **(B)** of the different strains. Same letters on top of each bar plot are not significantly different (LSD, *p* > 0.05).

The number of nodules was significantly higher (*p* ≤ 0.05, LSD) on seedlings infected by strain Lb8, as compared to the remaining isolates (Figure 1A). No significant difference in the diameters of nodules induced by the other four isolates was observed (*p* > 0.05, LSD). The efficiency of nitrogen fixation was significantly higher (*p* ≤ 0.05, LSD) for nodules induced by strain Lb8 than those induced by L1, L3, L4, and Ls1 (Figure 1B). The average length of gram-negative rod-shaped form cells (Supplementary Figure 3A) was 1.43 ± 0.44 μm long (Supplementary Figure 3B), 1.45 ± 0.6 and 1.21 ± 0.26 μm, for Lb8, Ls1, and L1, respectively.

**Figure 2:**
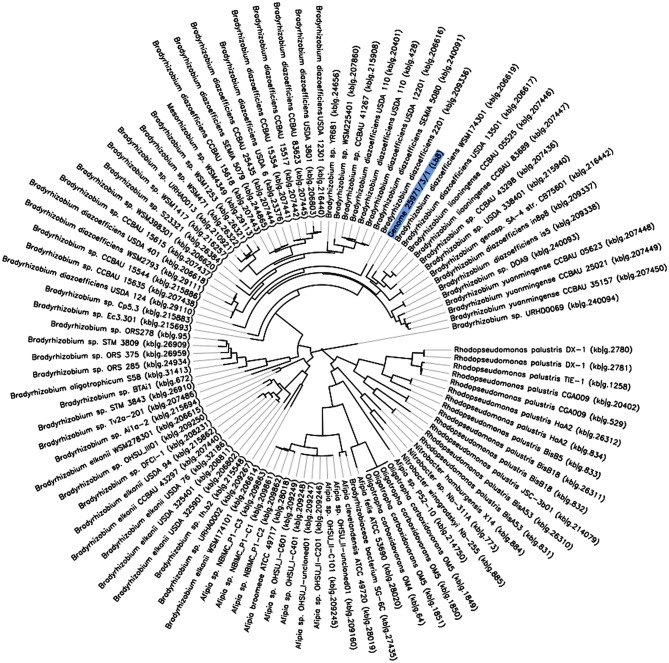
The phylogeny of *Bradyrhizobium* sp. Lb8 in comparison to a set of closely related genomes selected from all public KBase genomes. The phylogenetic tree was constructed using FastTree. The genome of *Bradyrhizobium* sp. Lb8 is highlighted in blue.

Owing to the relatively fast-growing nature, and its ability to induce higher number of nodules with nitrogenase activity, the Lb8 isolate was selected for whole genome sequencing. Based on a 16S rRNA gene sequence analysis, Lb8 was most related to *Bradyrhizobium kavangenese* isolated from the nodules of cowpea (Grönemeyer et al., 2015), with a sequence identity of 99%. It was clustered within a group of non-photosynthetic *nod* gene-containing bradyrhizobia, which included *B. diazoefficiens*, and was separated from those of photosynthetic *nod* gene-lacking strains, such as *Bradyrhizobium* sp. ORS278 (host: *A. sensitive* in Africa) and *Bradyrhizobium* sp. BTAi1 (host: *A. indica* in North America) (Giraud et al., 2007; Figure 2). No nodule was observed on soybean cultivars inoculated by Lb8 by 20 DAI indicating this strain may be specific to peanut.

### Genome Sequence of Lb8 Strain

**Figure 3:**
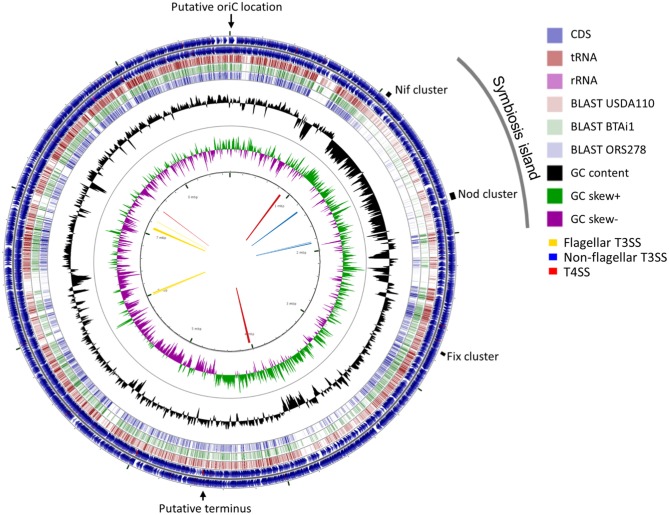
A circular representation of the chromosome of *Bradyrhizobium* sp. Lb8. The outermost and the second circle show the positions of the putative protein-encoding genes in clockwise and counter-clockwise directions, respectively. The third, fourth, and fifth circles from the outside represent BLASTn comparisons with *Bradyrhizobium* strains USDA 110^T^, BTAi1, and ORS278, respectively (*E* < 10^−10^). The innermost circle shows GC skew (higher than average GC content in green, lower than average in purple) and the second innermost circle shows the GC content of the entire sequence. Markings inside the innermost circle represent genome positions in Mb. Arrows in the center represent clusters of protein secretion systems. The positions of the putative replication origin, putative replication terminus, symbiosis island, and nitrogen fixation genes are shown outside of the outermost circle.

A total of 377,698 raw sequences, with approximately 2.36 Gb size, were obtained by PacBio sequencing. The mean read length was 6,253 bp, and the most extended read was 39,742 bp. The final genome assembly consisted of one circular chromosome of 8,718,147 bp with 63.14% GC content. The GC content was unevenly distributed in several locations. A shift of GC skew in two regions of the genome at coordinates 17 Kb and 4.7 Mb (Figure 3, the innermost circle) were assigned as a putative *oriC* location and terminus location (Supplementary Figure 4), respectively. No plasmid sequence was detected in the PacBio sequences obtained. The Lb8 genome was mostly collinear with *B. diazoefficiens* USDA 110^T^ except at the site of the symbiosis island (SI) (Supplementary Figure 5).

### Genome Annotation

A total of 8,433 protein-encoding genes (PEG)s were predicted, and 4,915 genes with clear known functions were classified into 27 categories, each with different biological roles, according to classic RAST (Table 1; Aziz et al., 2008). The average gene density was one gene per 1,027 bp. A total of 54 RNAs (non-PEG) were detected in the genome, including an rRNA gene cluster containing the 5S, 23S, and 16S rRNA genes (at coordinates 7,959,101–7,964,425 of the genome), and 51 tRNA genes scattered throughout the genome, for transferring the 20 standard amino acids.

**Table 1:** Number of identified protein encoding genes of known functions in *Bradyrhizobium* sp. Lb8 that are present in different subsystems according to the RAST server subsystem classification (Aziz et al., 2008).

**S. No**	**Subsystem category**	**Count**
1	Cofactors, vitamins, prosthetic groups, pigments	406
2	Cell wall and capsule	168
3	Virulence, disease and defense	129
4	Potassium metabolism	14
5	Photosynthesis	0
6	Miscellaneous	92
7	Phages, prophages, transposable elements, plasmids	2
8	Membrane transport	393
9	Iron acquisition and metabolism	4
10	RNA metabolism	186
11	Nucleosides and nucleotides	130
12	Protein Metabolism	309
13	Cell division and cell cycle	32
14	Motility and chemotaxis	188
15	Regulation and cell signaling	134
16	Secondary metabolism	6
17	DNA metabolism	122
18	Fatty acids, lipids, and isoprenoids	290
19	Nitrogen metabolism	84
20	Dormancy and sporulation	5
21	Respiration	251
22	Stress response	202
23	Metabolism of aromatic compounds	130
24	Amino acids and derivatives	761
25	Sulfur metabolism	132
26	Phosphorus metabolism	60
27	Carbohydrates	685
Total		4,915

A total of 84 PEGs were categorized into nitrogen metabolism (Table 1). Out of these, 11 were involved in cyanate hydrolysis, 20 were involved in nitrogen fixation, one in nitrosative stress, 18 in nitrate and nitrite ammonification, 16 in ammonium assimilation, and one was an amidase with predicted urea and nitrile hydratase functions. Seventeen PEGs were involved in denitrification. A region of 1,015 Kb (1,170,906–2,186,497) had consistently lower GC content (59.22%) compared to the average GC content (63.14%) of the whole genome. This region hosted two clusters of nitrogen fixation related genes and was termed a “symbiosis island” (SI). A met-tRNA gene was identified at one end of the region (2,185,706–2,185,781). Of the 711 putative PEGs assigned to the SI, 455 (64%) showed sequence similarity to genes of known functions and 18 PEGs were related to nitrogen metabolism.

The Lb8 genome possessed both Type III secretion systems (T3SS) and Type IV secretion systems (T4SS). Three clusters of non-flagellar T3SS (with 12, 5, and 4 genes, respectively), four clusters of flagellar T3SS (with 7, 7, 27, and 5 genes, respectively), and three clusters of P-type T4SS (with 16, 13, and 4 genes, respectively), were identified in the genome (Figure 3). T6SS secretion system was not identified in the Lb8 genome. T3SS secreted inner membrane proteins homologous to flagellar export components such as *yscQRSTUJL, spaRS, hrcRTUJV, excRT, epaR1, excRU, ssaUV, escRJV*, and *pscJ* which were located on the SI (peg.1219, peg.1224–peg.1226, peg.1230–peg.1232, peg.1234–peg.1235, and peg.1744–peg.1748). Similarly, homologs of T3SS, host injection protein (*yopB*), secretion bridge (*yscJ*), secretion cytoplasmic protein (*yscL*), and inner membrane channel protein (*lcrD, hrcV, escV*, and *ssaV*) were also included in the SI (peg.1757, peg.1759, peg.1762, and peg.1766). Homologs of T4SS (*virD4B11, tadAZC, cpaFEC*, and *rcpA*) were located outside the SI (peg.844, peg.1001, peg.1003–peg.1004, peg.1006, peg.3484–peg.3485, peg.3492, peg.3752, peg.31, and peg.1004), except one homolog, *rcpA*/*cpaE*, which was identified in the SI (peg.1236). The two paralogous *vir*D4 proteins (peg.844 and peg.3752) were 67% identical to each other and homologous to conjugal transfer protein *traG* in *B. diazoefficiens*. All the 17 *Bradyrhizobium* sp. with complete genome sequence available had clusters of flagellar T3SS while non-flagellar T3SS was present in 11 genomes, T4SS in 13 genomes, and T6SS was present in 8 genomes (Table 2).

**Table 2:** Number of clusters of different protein secretion systems in *Bradyrhizobium* strains predicted by T346 Hunter (Martínez-García et al., 2015).

**Strain**	**Version**	**NF-T3SS**	**F-T3SS**	**T4SS**	**T6SS**
*Bradyrhizobium* sp. BF49	LN901633.1		3		
*Bradyrhizobium* sp. BTAi1	CP000494.1		4	4	
*Bradyrhizobium* sp. CCGE-LA001	CP013949.1		3	1	
*B*. *diazoefficiens* E109	CP010313.1	1	4	2	1
*Bradyrhizobium* sp. G22	LN907826.1		4	1	1
*B. diazoefficiens* J5	CP017637.1	1	5	3	1
*B. icense* LMTR 13^T^	CP016428.1	1	3	2	
*B. diazoefficiens* NK6	AP014685.1	1	6	1	1
*Bradyrhizobium* sp. ORS 278	CU234118.1		3		
*Bradyrhizobium* sp. ORS 285	LT859959.1	1	3	2	
*Bradyrhizobium* sp. S23321	AP012279.1		4		
*B. oligotrophicum* S58	AP012603.1	1	3	1	
*B. diazoefficiens* SEMIA 5079	CP007569.1	1	4	3	1
*B. diazoefficiens* USDA 110^T^	CP011360.1	1	6	2	1
*B. diazoefficiens* USDA 122	CP013127.1	1	6		1
*B. diazoefficiens* USDA 6^T^	AP012206.1	1	4	2	1
*Bradyrhizobium* sp. Lb8		3	4	3	

*The number of clusters are presented under different protein secretion systems: NF-T3SS (= non-flagellar T3SS), F-T3SS (= flagellar T3SS), T4SS, and T6SS*.

Lb8 possesses nodulation genes including *nodA, nodC, nodD, nodI, nodJ, nodN, nodS, nodT, nodU, nodZ, nolA, nolY*, and *nolO*. Most of these genes were located in the SI except *nodN* and *nodZ*. Lb8 also possessed *fixA, fixB, fixC, fixJ, fixK, fixL fixR*, and *fixX* genes. A cluster of *fixJ* and *fixL* was located outside of the SI (coordinates of 2,967,208–2,969,821), while a paralogous *fixJ* (two-component nitrogen fixation transcriptional regulator) was identified inside the SI (coordinates 2,083,915–2,084,499). These genes shared 52% sequence identity with 99% query sequence coverage. A *fixC* gene (probable electron transfer flavoprotein-quinone oxidoreductase) was also identified in the genomic island (coordinates 1,264,204–1,265,511). A cluster of hydrogenase uptake (*hup*) genes *hupF*-*hypC-hoxLO-hyaE-hoxQV-hupK-hypABFCDE* was located on the SI (coordinates 1,214,329–1,227,503). These *hup* genes reduce energy loss associated with symbiotic nitrogen fixation and contribute to increased efficiency of nitrogen-fixation (Albrecht et al., 1978; Evans et al., 1987). Genes related to flagella formation (*fliJRQEPMLXIDKFGN, flhAFB, flgCBFGAHJLKED, flbT*, and *flaF*), including flagellar motor rotation genes (*motAB*) were found in the Lb8 genome. Lb8 had periplasmic nitrate reductase component genes, *napE* and *napD* (coordinates 7,255,262–7,255,773) and *norEQD* (nitric oxide reductase activation protein) formed a cluster (coordinates 3,452016–3,457,609). A total of 21 genes were annotated as putative transposases and eight of them were insertion sequences (ISs), though none of them were classified in the subsystem related to transposable element by RAST due to the limited curation of the RAST system (Table 1, Aziz et al., 2008). Homologs of *trbBCDEJLFGI*, which are essential for conjugative transfer of Ti-plasmid in *A. tumefaciens* (Li and Everhart, 1998), were found in two clusters (4,041,083–4,050,188 and 885,151–894,557). The first cluster included one T4SS cluster. A cluster of *trbIGFL* was also identified at another site of the genome (coordinates 7,430,109–7,434,552) showing gene duplications.

### Phylogenetic Analysis

A phylogenetic tree based on 49 highly conserved Clusters of Orthologous Groups (COG) placed *Bradyrhizobium* strain Lb8 with non-photosynthetic *nod* gene-containing bradyrhizobia (Figure 2) closely related to *B. diazoefficiens* WSM174301, which was isolated from soybean root nodules. *B. diazoefficiens* USDA 110^T^ from soybean root nodules was clustered in a separate node. A further phylogenetic analysis, based on concatenated *nod* genes, suggested that strain Lb8 was closely related to strains USDA 110^T^, SEMIA 5079, and USDA 6^T^ (Supplementary Figure 6).

### Comparative Genomics

The analysis of average nucleotide identity (ANI) between *Bradyrhizobium* sp. Lb8 and 17 other *Bradyrhizobium* strains with complete genome sequences showed that *Bradyrhizobium* sp. Lb8 represented a novel species (ANI < 95%, Supplementary Table 2). The closest fully assembled genome was *Bradyrhizobium* sp. CCGE-LA001 (ANI = 88.85%).

**Figure 4:**
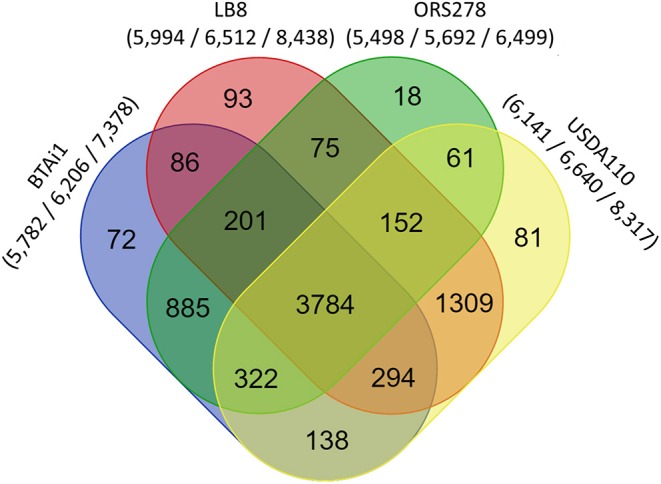
Comparative genomics analysis of *Bradyrhizobium* strains Lb8, USDA 110^T^, BTAi1, and ORS278. An oval represents each genome, and the number of shared and unique genes are shown by overlapping and non-overlapping parts of the ovals (OrthoMCL 2.0.7, *E* < 10^−5^). The numbers under species names indicate the number of gene families, the gene number in the families, and the total number of genes (protein sequences) input into OrthoMCL.

A reciprocal BLAST comparison was conducted between Lb8, and three other nodulating *Bradyrhizobium* strains with complete genome sequence availability, namely, USDA 110^T^, ORS278, and BTAi1. Overall, 5,712 (68.6%), 4,534 (54.4%), and 4,409 (52.9%) genes showed the same best reciprocal hit between Lb8 and USDA 110^T^, BTAi1, and ORS278, respectively. To further evaluate the relationship between Lb8 and the three other strains, OrthoMCL was used to cluster the orthologous groups (gene families) among the four strains. In total, 7,571 orthologous groups were clustered from 25,050 protein sequences. These four strains shared 3,784 gene families. Among the grouped gene families, Lb8 shared 5,539 (92.41%) gene families with USDA 110^T^, followed by 84,365 (72.82 %) gene families with BTAi1, and 4,212 (70.27%) gene families with ORS278. There were 93 (1.55%) gene families corresponding to 298 protein sequences unique to Lb8 (Figure 4).

Out of the 152 genes related to nitrogen metabolism, 75 genes were present in all four strains (Supplementary Table 1) with 42 genes (56%) highly conserved across these four genomes (Supplementary Table 1, Supplementary Figure 7). Out of these 42 genes, 15 were related to nitrogen fixation, five were related to cyanate hydrolysis, one was amidase, five were related to nitrate and nitrite ammonification, five were related to ammonium assimilation, two were in the denitrifying reductase gene cluster, and the remaining nine genes were related to denitrification. In the Lb8 strain, genes related to nitrogen metabolism such as *cynR* (cyanate hydrolysis), *nifT*, and *ginE* (ammonia assimilation) were unique in comparison to other three *Bradyrhizobium* strains (ORS278, BTAi1, and USDA 110^T^).

**Figure 5:**
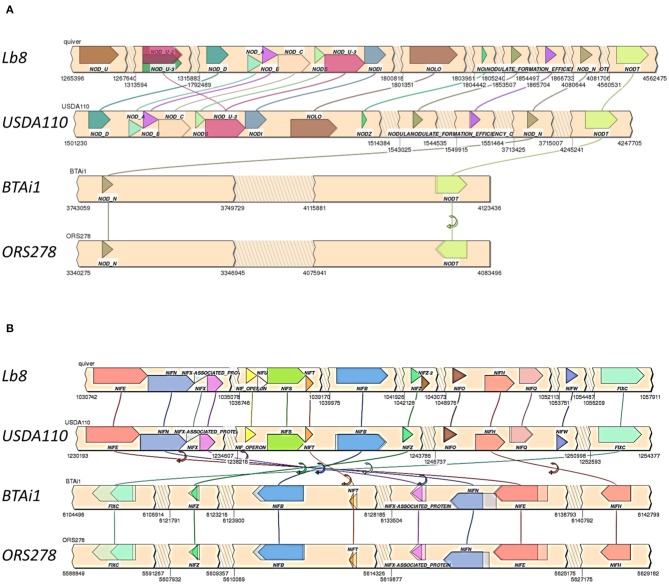
Synteny of clustered *nod* **(A)** and *nif* /*fix* **(B)** genes in Lb8, USDA 110^T^, BTAi1, and ORS278 genomes. Genes are color coded with labels listed above or below the genes. Gene direction is indicated as pointed ends. Flip arrows indicate a change in direction between genomes. Jagged edges indicate skipped sections.

Homologs of *nodQ, nodM, nodI, nodD1, nodD2, nodN, nodV*, and *nodW* were present in all the four genomes compared. *nodY* gene did not have homologs in Lb8, ORS278, and BTAi1 but was present in USDA 110^T^. *nodS* and *nodU* were present in both Lb8 and USDA 110^T^ but were absent in ORS278 and BTAi1. *nod* genes present in Lb8 were collinear with those in USDA 110^T^ (Figure 5A). Three copies of the *nodU* genes were present in the Lb8 genome. Interestingly, gene homologs of *nodN* and *nodT* were found in all four genomes analyzed. *nodT* was inverted in ORS278. Similarly, *nif* and *fix* genes were collinear between Lb8 and USDA 110^T^ (Figure 5B). *nif* and *fix* genes were also collinear between BTAi1 and ORS278 genomes. However, *nif* and *fix* genes were inverted in the BTAi1 genome, and rearrangement of genes was seen on the BTAi1 genome as compared to the USDA 110^T^ or Lb8 genome.

## Discussion

Nitrogen is an essential and often growth-limiting nutrient for plants (Gresshoff and Ferguson, 2017). The association of nitrogen-fixing rhizobia with legumes is a sustainable and environmentally friendly alternative to synthetic nitrogen fertilizers (Gresshoff and Ferguson, 2017). Many *Bradyrhizobium* sp. can nodulate peanuts, where a high degree of correlation exists between nitrogenase activity and shoot weight, nodule number, nodule mass, and total nitrogen (Wynne et al., 1983; Nigam et al., 1985; Li et al., 2015, 2019). Approximately 90% of the world peanut crop is produced in developing countries where the pod yield is around four times less than that in developed countries (Boogerd and Van Rossum, 1997). Therefore, the selection of appropriate rhizobial strains could lead to an improvement in nodule number, shoot weight, and plant color (Wynne et al., 1983). This will eventually increase the amount of nitrogen available for the plants, subsequently improving pod yield. Moreover, nodulation in peanuts occurs via a “crack entry” mechanism (Howieson and Dilworth, 2016), which is believed to be evolutionarily more ancient than infection via root hairs (Sprent, 2007). Therefore, unraveling the mysteries of this ancestral form of colonization would increase our knowledge in identifying suitable symbiosis clusters that potentially could be transferred to other non-legume crops.

A high level of diversity and heterogeneity exist in peanut symbionts. Several fast-growing endophytic isolates of *Gammaproteobacteria* that were unable to elicit root nodules were recovered from peanut nodules in Argentina (Ibáñez et al., 2009), as well as some slow-growing *Bradyrhizobium* isolates that have been characterized (Muñoz et al., 2011). However, complete genomes of root-nodulating bacteria that live in symbiosis with peanuts are not available. On the other hand, *B. diazoefficens* USDA 110^T^ that fixes nitrogen in economically important crops such as soybean, has been completely sequenced (Kaneko et al., 2002). Other *Bradyrhizobium* strains with complete genome sequences include *Bradyrhizobium* sp. BTAi1 and *Bradyrhizobium* sp. ORS 278, which form nodules on *Aeschynomene* (Giraud et al., 2007). Complete sequences of *Bradyrhizobium* sp. CCGE-LA001, which was obtained from a wild *Phaseolus* sp., and non-nodulating isolate, *Bradyrhizobium* sp. S23321, which was isolated from paddy fields, are also available (Okubo et al., 2012b; Rogel and Moral, 2016). Limited knowledge on the interaction of peanut with rhizobia restricts our ability to fully utilize the nitrogen-fixing capacity of rhizobia in crack entry species. Therefore, this study was initiated to sequence the whole genome of an efficiently growing *Bradyrhizobium* strain capable of inducing peanut root nodules and to characterize the strain.

### Sequence Features of the Lb8 Genome

An isolate, Lb8, was selected for whole-genome sequencing because of its relatively fast growth rate and high nitrogenase activity compared to the other isolates from peanut nodules. Lb8 had identical 16S rRNA sequence to Ls1, but they had different growth curves and nitrogenase activity. Despite having identical 16S rRNA, bacteria could show variable biosynthetic properties. For example, multiple strains in *Brevundimonas alba* with identical 16S rRNA sequence had different growth rate and cell yield (Jaspers and Overmann, 2014). Similarly, various species in *Streptomyces* that had identical 16S rRNA sequence showed different phenotypic and morphologic properties (Antony-Babu et al., 2017). In our research, the differences in the growth curve of these isolates could be explained by variations in other parts of the genome. Phylogeny results from 16S rRNA and housekeeping genes suggested that the Lb8 genome resembled more closely the non-photosynthetic *nod* gene-containing bradyrhizobia than the photosynthetic *nod* gene-lacking bradyrhizobia. This was corroborated with RAST annotations on whole genome sequencing data, where we could not find any genes related to photosynthesis (light-harvesting complexes, photosynthesis, or electron transport and photophosphorylation). Although symbiotic genes do not offer taxonomic information because they are located in easily interchangeable elements (Chahboune et al., 2011), a phylogenetic tree based on *nod* genes showed that Lb8 belongs to the same symbiovar as strains SEMIA 5079, USDA 110^T^, and USDA 6^T^. ANI values of Lb8 compared with other sequenced *Bradyrhizobium* genomes indicated that *Bradyrhizobium* sp. Lb8 was a novel species that fixes nitrogen in peanut nodules.

We didn't find any plasmid sequences in the PacBio sequences of the DNA sample, which was prepared from the ChargeSwitch gDNA mini bacteria kit to target the genomic DNA of the bacteria. In addition, bradyrhizobia typically have no plasmid, which is a standard feature of bradyrhizobia (MacLean et al., 2007). Plasmids are more common in fast-growing rhizobia such as *Sinorhizobium fredii* (Scholla and Elkan, 2009). The symbiotic island in Lb8 was integrated in its genome, which is a typical feature of non-photosynthetic *Bradyrhizobium* strains, such as USDA 110^T^. Even though the Lb8 genome shared 92% of the gene families with USDA 110^T^, it showed several key differences. The genome of *Bradyrhizobium* sp. Lb8 was ~400 kb smaller than that of USDA 110^T^ and it possessed a much larger symbiotic island than USDA 110^T^. Unlike USDA 110^T^, Lb8 did not possess homologs of *nodY* gene and the T6SS was not present in Lb8. Presence of genes like *cynR, nifT*, and *ginE* in Lb8 make it unique. Lb8 lacks nitrous oxide reductase genes like *nosDXFRY* that are present in USDA 110^T^. Lb8 had only one cluster of *nif* genes whereas in USDA 110^T^ they were present in two clusters.

### Symbiosis Island

Acquisition of the SI converts a saprophyte into a symbiont. Therefore, the island is likely to contain other genes that contribute to successful plant-microbe interactions, in addition to genes required for nodule formation and symbiotic nitrogen fixation (Sullivan and Ronson, 1998). SIs were identified in *M. loti* ICMP3153 and *M. japonicum* MAFF303099 as DNA segments of 500 to 611 Kb with low GC content and flanked by a complete and partial phe-tRNA gene (Sullivan and Ronson, 1998; Hubber et al., 2004). A split SI of 410 Kb plus 6 Kb was identified in a 680 Kb DNA region of low GC content in USDA 110^T^ (Kaneko et al., 2002). Sequence analysis of the Lb8 genome also revealed the presence of a 1,015 Kb genomic island with relatively low GC content, including a complete 777 Kb symbiotic DNA region. Genomic islands are usually integrated at the tRNA loci, with an integrase gene at the 5′ junction (Hacker and Kaper, 2000; Zhang and Zhang, 2004). In *M. loti*, a partially duplicated phe-tRNA gene was identified while a partially duplicated val-tRNA gene was identified in USDA 110^T^. The presence of a partially duplicated met-tRNA gene and a putative integrase gene near the end of this island in Lb8 reinforces the presence of an SI in the genome. The association of SIs with a phage-related integrase implies that SIs may have originated from an ancient integration of bacteriophage. Most genomic islands have mobile genes like transposase and integrase (Hacker and Kaper, 2000). It was remarkable that 16 out of the 21 transposases detected (76%) and six out of 16 integrase genes (38%) were located in the presumptive SI in Lb8 with low GC content. Therefore, we hypothesize that an ancient SI was transferred by horizontal gene transfer, which is well-known to contribute to microbial evolution (Hacker and Kaper, 2000).

### Nitrogen Metabolism-Related Genes

Genes related to different steps of the symbiotic nitrogen fixation process were identified in the Lb8 genome. Specifically, *cynR* (cyanate hydrolysis), *nifT*, and *ginE* (ammonium assimilation) were unique in comparison to the other *Bradyrhizobium* strains such as ORS278, BTAi1, and USDA 110^T^. Although the exact function of *nifT* has not been identified, it has been suggested to have a limited role in nitrogenase maturation in *Klebsiella pneumoniae* (Simon et al., 1996).

In most rhizobia, *nod* genes are essential for Nod factor biosynthesis and transportation. Bacterial mutants defective in Nod factor production can no longer associate with host legumes, and legume mutants that are defective in the perception of Nod factors can no longer host the bacteria (Oldroyd, 2013), with few exceptions. In peanuts, *nod*C gene was essential to induce cortical cell division leading to formation of nodule primordium. The absence of the gene, however, did not affect the bacteria's ability to colonize peanut roots (Ibáñez and Fabra, 2011). *nodD* is essential for the expression of the other *nod* genes (Stacey et al., 1995). *NodD1* appears to be the only functional *nodD* gene in *B. diazoefficiens*, and its activation is dependent on the presence of isoflavones (Stacey, 1995). *NodV* and *nodW* may also be able to activate *nod* gene expression (Stacey, 1995). In the Lb8 genome, *nod* genes were present in two clusters. Cluster I contained most of the *nod* genes (*nodU*_1_*U*_2_*DABCSU*_3_*I-nolO-nodZ-nfeC*). *NodT* and *nodN* were found in a separate cluster in the symbiosis island. The presence of *nodABC* genes, which are required for the synthesis of the lipo-chitooligosaccharidic backbone, suggests that Lb8 nodulates peanuts in a Nod factor-dependent manner, but it is also possible that these genes have been retained to colonize alternative legume hosts. Photosynthetic *Bradyrhizobium* strains, such as BTAi1 and ORS278, without the *nodABC* genes required for Nod factor biosynthesis can still colonize their host legumes (Giraud et al., 2007), indicating the possibility of an alternative pathway to initiate symbiosis (Masson-Boivin and Sachs, 2018).

In addition to the *nod* genes, *nif* and *fix* are essential genes involved in nitrogen fixation among *Bradyrhizobium* species and other rhizobia. *nif* genes found in bradyrhizobia have similar function and structure with the related genes found in *Klebsiella pneumoniae*, a free-living diazotroph (Fischer and Hennecke, 1987; Hennecke, 1990). *nifW* and *nifZ* genes are involved in MoFe protein maturation, and their requirement varies significantly among diazotrophs (Li et al., 2016). *nifBENH* are involved in the synthesis of FeMo-co from iron, sulfur, molybdenum, and homocitrate under reducing conditions (Rubio and Ludden, 2008). *nifQ* is an iron–sulfur protein with a redox-responsive [Fe–S] cluster and is also a molybdoprotein that donates molybdenum for FeMo-co synthesis in the simultaneous presence of *nifH* and *nifEN* (Rubio and Ludden, 2008). *nifS* directs the assembly of *nifU*, and with *nifB* sequentially provides the iron–sulfur core while *nifX* binds a variety of structurally related factors (Rubio and Ludden, 2008). *Fix* genes are essential for symbiotic nitrogen fixation. In *B. diazoefficiens* strain USDA 110^T^, the *nif* and *fix* genes were found in at least two different clusters on the chromosome. Cluster I included most of the *nif* genes. Cluster II contained three *fix* genes (Hennecke, 1990). However, in Lb8, *nif* genes were present in a single cluster, including *nifWQHOZBTSUXNE*. Presence of this gene cluster shows an active role of Lb8 in nitrogen fixation. One *fix* gene (*fixC*) was also detected in the cluster. The genome also contained *fixJ*, which controlled symbiotically important genes other than those whose expression was dependent on the NifA protein (Anthamatten and Hennecke, 1991). Nif A protein activates *nif* genes in *B. diazoefficiens* strain USDA 110^T^ and is sensitive to oxygen (Fischer and Hennecke, 1987). The presence of the hydrogenase gene cluster increases the efficiency of nitrogen fixation because Ni-Mo hydrogenase mediates uptake of dihydrogen via nitrogenase (Kaneko et al., 2002). Plants with nodules containing hydrogenase systems were shown to fix more nitrogen and produced higher dry weights than plants nodulated with strains lacking hydrogen uptake genes (Albrecht et al., 1978). The presence of *hup* genes in Lb8 might promote higher efficiency of nitrogen fixation.

Lb8 possesses a *nirV* gene, which is required for nitrite reduction and is found in bacteria with copper-containing nitrite reductases (Jain and Shapleigh, 2001). A copper-containing nitrite reductase was observed immediately upstream of the *nirV* gene in Lb8. Thus, Lb8 should have the capacity to reduce nitrite. Putative products of periplasmic nitrate reductase genes are likely to be involved in denitrification from nitrate to N_2_ (Black et al., 2012). A homolog of a *tfdB*-like gene (2,4-dichlorophenol 6-monooxygenase), which is implicated in the degradation of 2,4-D (2,4-dichlorophenol; Vallaeys et al., 1999), was also found in Lb8.

### Protein Secretion Systems

Protein secretion systems play a crucial role in the interaction of bacteria and their eukaryotic hosts (Eichinger et al., 2016). T3SSs are involved in host colonization, virulence, and symbiotic interactions and they can be horizontally transferred (Poggio et al., 2007; Cole et al., 2017; Levy et al., 2018). T4SSs are involved in bacterial conjugation, DNA uptake and release, and effector translocation (Fronzes et al., 2009). Lb8 possesses both T3SS and T4SS genes. Interestingly, the genes for the non-flagellar T3SS were located on the symbiosis island, while the genes for the flagellar T3SS and the T4SS were located outside the symbiosis island. This feature is similar to USDA 110^T^ (Kaneko et al., 2002), except that USDA 110^T^ also possesses a single T6SS cluster outside the symbiosis island (Banerjee et al., 2019). USDA 110^T^ has been classified as “Root Nodular II” based on its presence of T3SS, T4SS, and T6SS secretome (Banerjee et al., 2019). On the other hand, the absence of T6SS and the presence of T3SS and T4SS makes Lb8 closer to the “Root nodular I” type genomes. Flagellar T3SSs drive cell mobility while non-flagellar T3SSs deliver bacterial effectors into host cells via channels that cross the bacterial envelope and the host cell membrane to establish trans-kingdom interactions (Abby and Rocha, 2012; Deng et al., 2017). Rhizobial T3 effectors suppress plant defense responses against rhizobia and promote symbiosis-related processes (Staehelin and Krishnan, 2015). Type IV effectors secreted by the T4SS in *M. japonicum* MAFF303099 promoted nodule formation on its host, *Lotus corniculatus* (Hubber et al., 2004; Martínez-Hidalgo et al., 2016). The T4SS and T3SS may be involved in fine-tuning of host-specific nodulation in peanuts. In *M. japonicum* MAFF303099, genes for both T3SS and T4SS were located inside the symbiosis island (Sullivan and Ronson, 1998; Martínez-Hidalgo et al., 2016). Presence of the flagellar T3SS in all 17 *Bradyrhizobium* strains analyzed in this study indicates that the flagellar T3SS might be an integral component of symbiosis.

In summary, current study revealed a whole genome analysis of Lb8, a native *Bradyrhizobium* strain isolated from peanut nodules. The availability of this genome will facilitate the understanding of how rhizobia work and interact with peanut. To ultimately clarify the unique mechanisms of crack entry, it will be necessary to characterize the infection path of rhizobia into peanut roots. While previous studies have shown that Nod factors might not be needed for colonization of peanut roots, Nod factors are known to be involved in the initiation of cortical cell division (Ibáñez and Fabra, 2011). The *nod* genes identified in this study could also shed light on subsequent studies on the requirement for Nod factors in peanut–rhizobium interactions. Crack entry is believed to be evolutionarily ancient and is the simplest entry method, so it holds high promise to be used as a donor in extending the nitrogen fixation symbiosis to important non-legume crops. We want to emphasize that *Bradyrhizobium* sp. Lb8 could be a model microorganism for nodulating soil bradyrhizobia for crack entry species, providing a valuable tool for experiments in genetics, physiology, and ecology of such species. Additional investigations into the crack entry mechanism will not only impact biotechnological applications and enhance our comprehension of plant–microbe interactions but may also be vital to illustrate the evolution of symbiosis.

## Materials and Methods

### Strain Isolation

Two cultivated peanut lines, Tifrunner and UF 487A were grown in the field of the Plant Science Research and Education Unit of University of Florida at Citra, FL, which was inoculated with a commercial peanut inoculum. Nodules from 30-day-old plants were harvested from these two peanut lines. A total of 20 individual nodules were obtained from 10 plants of two peanut lines, Tifrunner and UF 487A. The procedure used for rhizobia isolation followed the method described previously (Taurian et al., 2002, 2006) with some modifications. In brief, the detached nodules from roots were surface sterilized by immersing in 95% ethanol for 30 s and in 0.1% HgCl_2_ for 4 min, followed by washing six times with sterile distilled water. The surface-sterilized nodules were individually crushed in a drop of sterile water. The suspension of the crushed nodules after centrifugation was streaked on 20 yeast extract mannitol (YEM) agar plates with one nodule suspension per plate and incubated at 28°C for 7 days (Vincent, 1970). Strains were streaked to single colonies three times to ensure that it was a pure culture. Colonies of different sizes and colors were representatively picked for colony PCR to amplify 16s rRNA gene.

### Colony PCR and Sequencing

For colony PCR, a pair of primers designed from 16S rRNA gene were synthesized by Invitrogen: F27: 5′-AGAGTTTGATCATGGCTCAG-3′, and R1541: 5′-AAGGAGGTGATCCAGCCGCA-3′ (Weisburg et al., 1991). A total volume of 20 μL reaction mixture was composed of the single colony of bacteria, 2 μL of 10× buffer [0.1 mol/L Tris-HCl (pH = 9.0), 0.5 mol/L KCl, 7.5 mmol/L MgCl_2_, 0.1% Triton X-100], 5 μmol of each primer, 2.5 mmol/L each of deoxynucleotide triphosphates (dNTP), and 1 unit of *Taq* DNA polymerase (Promega). Amplification was carried out on the program for the initial denaturing step with 94°C for 5 min, followed by 35 cycles for 30 s at 94°C, 45 s at 55°C, 1 min at 72°C, with a final extension at 72°C for 10 min. The amplicons were separated on a 1.5% agarose gel for 1 h at 150 V. Selected representative PCR products were purified by using a QIAgen PCR purification kit (Cat. No. 28104) according to the manufacturer's protocol and then sequenced by the Sanger method at the Interdisciplinary Center of Biotechnology Research at the University of Florida. The trimmed clean sequences were BLASTed against the NCBI GenBank nucleotide database to identify the best hit sequences with known annotation. The taxonomy-confirmed colonies were picked and stored in 20% glycerol solution at −80°C.

### Gram Staining, Morphological Observation, and Growth Curve Characterization

Gram staining was done following the standard protocol (Christopher and Bruno, 2003). The slides were observed under an Olympus BX41 microscope (Olympus America Inc., Melville, NY, USA). Scanning electron microscopy observation was performed according to published protocol (Mazia et al., 1975). Briefly, cultures were deposited onto poly-L-lysine treated 0.2 μm Millipore filters and fixed by immersion into 4% (v/v) paraformaldehyde, 2.5% (v/v) glutaraldehyde in 0.1 M cacodylate buffer, pH 7.3 (Electron Microscopy Sciences). Filters containing cells were rinsed twice in 0.1 M cacodylate buffer, water washed thrice, and dehydrated through a gradient of ethanol (25, 50, 75, 95, 100%). Dehydrated cells were critical point dried (Autosamdri-915, Tousimis) and mounted with a carbon adhesive tab and graphite paste onto a 12 mm aluminum SEM stub (Ted Pella, Inc.). The samples were further rendered conductive by sputter coating with Au/Pd and argon gas (DeskV, Denton Vacuum) and imaged on a Hitachi SU5000 Schottky Field-Emission Variable Pressure SEM (Hitachi High Technologies, America). The cell size was measured on 20–30 randomly selected individual cells.

To evaluate the growth rate of each isolated bacterial strain, we streaked a line on YEM medium in stock solution and cultured at 28°C until a colony was seen after about 7 days. Single colonies were picked with tips and kept in a 50 ml tube with 30 ml YEM medium. Each strain had at least three replicates. The cultures were diluted to 2x, 4x, and 10x on day two, three, and four, respectively. The tubes were shaken for 28 days, and the OD_600_ value of each strain solution was measured at the same time every day to evaluate the growth rate of strains.

### Nodule Formation Test

To test whether the isolated strains can infect peanut and form functional nodules, each isolated rhizobial strain was inoculated into 200 ml YEM broth in a shaking incubator at 28°C. When the OD_600_ value reached 0.05–0.1, the liquid cultures were spun down at 4,000 × *g* for 15 min. Bacterial cells were then washed with sterile water and re-suspended to OD_600_ = 0.05–0.1, with ~10^6^ cells per ml as inoculum.

Seeds of the cultivated peanut genotype Tifrunner and soybean cultivars, Bennings and Boggs, were sterilized in 0.1% HgCl_2_ solution for 7 min, and then washed for three times using ddH_2_O for 5 min each time. Sterilized seeds were immersed in a 500 ml glass flask for 2 days and then transferred to germination box with germination paper soaked with ddH_2_O and incubated at 25°C. After 4–5 days, germinated seeds were transferred to a growth bag (Ziploc plastic bag with filter paper) with 40 ml of 25% Hoagland's solution without nitrogen and grown in a growth chamber with 12 h light/12 h dark cycles and at 25°C. The growth bags were supported upright on a board by clippers. When the root length was ~6 cm, plants were selected to be inoculated by each rhizobial isolate separately with three biological replicates. For rhizobial inoculation, a 1-ml bacterial suspension (OD_600_ = 0.05–0.1) was applied to the surface of the root in the growth bag for inoculation. Water and Hoagland's solution were used as controls. Nodules were counted and then harvested for weight and size measurement and dissection and color observation at 2 weeks after inoculation. The diameter of mature nodules was evaluated by measuring the total diameters of 5 mature nodules on each plant using a Digital Caliper. All analysis and plots were done in software R, v 3.4.4 (http://www.R-project.org/) unless noted otherwise.

### Nitrogenase Activity Measurement

Seeds of cultivated peanut genotype Tifrunner were inoculated with different strains to test nitrogenase activities of these strains during symbiosis by acetylene reduction assay (ARA) (Hardy et al., 1973). The inoculation procedure was the same as described above. At 12 days post-inoculation, primary roots with nodules inoculated by different strains were cut and put into three 140 ml bottles with a rubber stopper. Each bottle had 2–3 roots with nodules. From each bottle, 10 ml air was removed and replaced with the same amount of acetylene (10 ml) by syringe injection. The bottles were then sealed and incubated at 28°C for 4 h. After the reaction, 1 ml of air from each bottle was taken out by syringe, and the amount of ethylene formed was measured by using a Gas Chromatographer (5890 Series). After ethylene measurement, nodules were cut from roots in each bottle to get fresh weight. Nitrogenase activity (nmol/mg/min) was calculated using the following formulae:

(1) $$\begin{array}{l} \text{amount of ethylene in 1 ml sample }\left(\%\right)=\;\frac{\text{amount of standard ethylene }\left(\%\right)\;\times \text{ peak area in sample}}{\text{peak area of standard ethylene}} \end{array}$$

(2) $$\begin{array}{l} \text{total ethylene formation }\left(\text{L}\right)=\text{amount of ethylene in }1\text{ ml sample }\left(\%\right)\times \text{volume of bottle }\left(\text{L}\right) \end{array}$$

(3) $$\begin{array}{l} \text{total ethylene formation }\left(\text{nmol}\right)=\frac{\text{total ethylene formation }\left(\text{L}\right)}{22.4\text{ L}}\times 10^{9} \end{array}$$

(4) $$\begin{array}{l} \text{nitrogenase activity }\left(\text{nmol}/\text{mg}/\text{min}\right)\;=\frac{\frac{\text{total ethylene formation }\left(\text{nmol}\right)}{\text{fresh weight of nodules }\left(\text{mg}\right)}}{\text{reaction time }\left(\text{min}\right)} \end{array}$$

### Sequencing and Data Analysis

Genomic DNA was extracted from a 50 ml liquid culture of a single colony using ChargeSwitch gDNA mini bacteria kit (Life Technologies) for whole-genome sequencing using the PacBio RS II system (Pacific Biosciences). An approximately 20 Kb insert PacBio library was constructed and sequenced in three SMRT cells. The PacBio reads were *de novo* assembled using the HGAP (Chin et al., 2013) protocol, which generated an initial assembly of 24 contigs with a total length of 11,944,080 bp. Plasmid Finder (v1.3) (Carattoli et al., 2014) was used to check for the presence of any plasmids. BLASR (Chaisson and Tesler, 2012) was used to map reads on contigs. The average depth per contig ranged from 43× to 69×, except for unitig_0, which had 403× coverage. Reads that mapped to unitig_0 (a contaminant contig which was closely related to *Microbacterium* sp. CGR1) were removed. Remaining reads were then extracted and used for assembly using the HGAP version 1 protocol under SMRT Analysis 2.1.1 and polished using Quiver (Pacific Biosciences). The contig was circularized using Circlator/1.5.1 (Hunt et al., 2015) and reverse complemented with EMBOSS/6.5.7 to get the final assembly. Annotation of the genome was done according to the RAST protocol (Aziz et al., 2008). NCBI-BLAST (https://blast.ncbi.nlm.nih.gov/Blast.cgi) was used to further identify any additional symbiosis related genes in Lb8 with default settings. A putative *oriC* was identified by using Ori-Finder (Gao and Zhang, 2008). rRNA genes were predicted using the RNAmmer 1.2 (http://www.cbs.dtu.dk/services/RNAmmer/) web server. Protein secretion systems were identified using T346 Hunter (Martínez-García et al., 2015).

### Genome Comparison and Ortholog Analysis

A circular genome map showing GC skew and GC content was created using CGview server with default parameters (Grant and Stothard, 2017). Putative orthologous genes were identified using bidirectional BLASTn comparisons with an E-value cutoff of 10^−20^. OrthoMCL software (Fischer et al., 2011) was used to identify orthologous gene groups between the rhizobial genomes being compared. Protein sequences of Lb8 were downloaded from the results of the RAST server, and the protein sequences of other genomes were downloaded from the NCBI website (BTAi1: NC_009485.1, USDA 110^T^: NZ_CP011360.1, and ORS278: CU234118.1). OrthoMCL analysis on these protein sequences was done by following “Basic Protocol 2” from (Fischer et al., 2011), with default parameters. An E-value of “1e^−5^” was used for all-versus-all BLASTp comparisons. Orthologous relationships were depicted in a Venn diagram.

Average Nucleotide Identity (ANI) between Lb8 and 17 *Bradyrhizobium* strains with complete genome sequences (Genbank ID: LT859959.1, CP013949.1, CP013127.1, CP016428.1, LN907826.1, LN901633.1, CP017637.1, CP011360.1, AP014685.1, CP010313.1, CP007569.1, AP012603.1, CU234118.1, CP000494.1, BA000040.2, AP012279.1, AP012206.1) was calculated using ANI calculator (Rodriguez and Konstantinidis, 2016).

### Phylogeny of Nodulation and Nitrogen Fixation Related Genes

Phylogenetic analysis was conducted using KBase (Arkin et al., 2016) with the “Insert Genome Into Species Tree” app that used 49 highly conserved Clusters of Orthologous Groups (COG) families to find matching corresponding sets of sequences for a specific genome. Sequences from the selected genomes were then inserted into the reference alignments, and closest neighbors were extracted and concatenated. After that, a tree was rendered using the FastTree 2 algorithm (Price et al., 2010). Phylogenetic analysis of *nod* genes, including *nodU*_1_*U*_2_*DABCSU*_3_*I-nolO-nodZ-nfeC-nodNT* in selected species, was constructed using Geneious v.10.2.3 (Drummond et al., 2011). A multiple alignment for each gene was done using ClustalW (Cost matrix–BLOSUM; Cap open cost−10; Gap extend cost−0.1). The alignments were concatenated, and the concatenated alignment was used to construct a phylogenetic tree (Alignment type–Global alignment with free end gaps; Cost matrix–BLOSUM62; Genetic Distance Model–Jukes-Cantor; Tree build Method–Neighbor-Joining; Outgroup–No Outgroup; Bootstrap−1000).

## Data Availability Statement

The whole genome sequence of *Bradyrhizobium* sp. Lb8 is available from RAST server with genome ID 6666666.283533.

## Author Contributions

JW conceived the research and designed the experiments. DP, FL, LW, MC, SM, ZP, and KK performed the experiments and analyzed the data. DP prepared the manuscript draft. JW, MC, and J-MA critically revised the manuscript. All authors approved the final version of the manuscript.

### Conflict of Interest

The authors declare that the research was conducted in the absence of any commercial or financial relationships that could be construed as a potential conflict of interest.

## Supplementary Material

Supplementary Figure 1 A growth curve of five peanut nodule isolates grown at 28°C in YEM medium.

Supplementary Figure 2 **(A)** Nodules formed on Tifrunner roots 13 days after inoculation by five different strains. **(B)** Root nodules showing active nodulation after 22 days of inoculation with the different strains.

Supplementary Figure 3 **(A)** A scanning electron micrograph of the three strains L1, Lb8, and Ls1. **(B)** Bar plots showing the length of the different strains (*n* = 44). Same letters on top of each bar plot are not significantly different (HSD, *p* > 0.05).

Supplementary Figure 4 A GC-skew plot showing the putative locations of the *oriC* and terminus in the *Bradyrhizobium* sp. Lb8 genome.

Supplementary Figure 5 A dot plot showing the similarity between *Bradyrhizobium* sp. Lb8 and *B. diazoefficiens* USDA 110^T^.

Supplementary Figure 6 Neighbor-joining tree based on concatenated sequences of *nod* genes present in *Bradyrhizobium* sp. Lb8 (*nodU*_2_*DABCSU*_3_*I-effC-nodZNT*) and closely related species within the genus *Bradyrhizobium*. The significance of each branch is indicated by a bootstrap value calculated for 1,000 subsets. Bar, 3 substitutions per 1,000 nucleotides.

Supplementary Figure 7 The number of nitrogen metabolism-related genes that are present in the different *Bradyrhizobium* genomes.

Supplementary Table 1 Nitrogen metabolism related genes in four *Bradyrhizobium* genomes. The numbers in the list refer to locus numbers on the RAST server. Numbers in column names after the species name refer to accession number (In Excel sheet).

Supplementary Table 2 ANI (%) values for comparison of *Bradyrhizobium* sp. Lb8 with 17 complete genomes of *Bradyrhizobium* (In Excel sheet).
