# Supplementary figures and images for: Isolation, Characterization, and Complete Genome Sequence of a Bradyrhizobium Strain Lb8 From Nodules of Peanut Utilizing Crack Entry Infection
Source: Front Microbiol. 2020 Feb 7;11:93. doi: 10.3389/fmicb.2020.00093 (PMC7020250; doi:10.3389/fmicb.2020.00093)

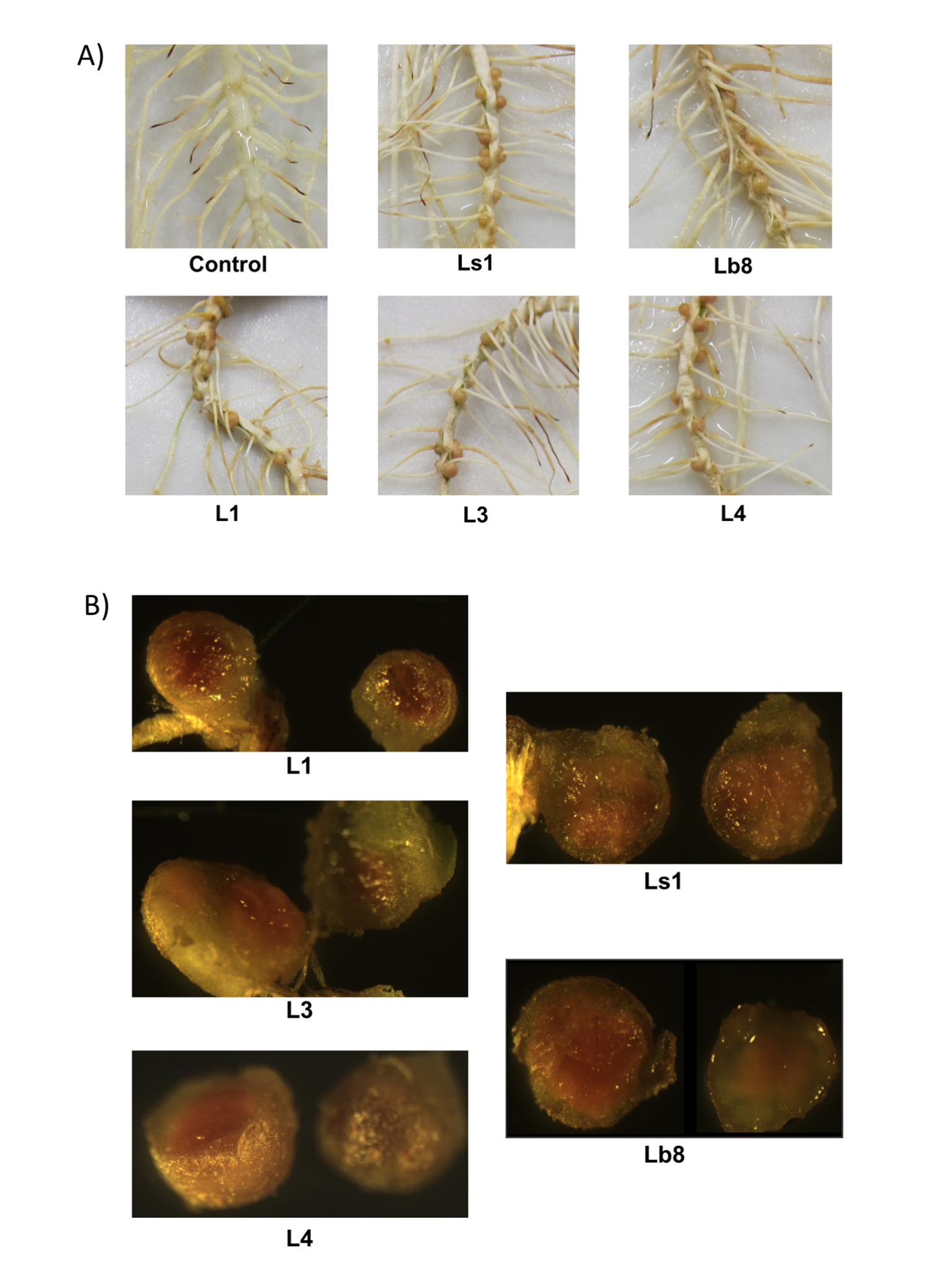

Supplement: Supplementary Figure 2 — (A) Nodules formed on Tifrunner roots 13 days after inoculation by five different strains. (B) Root nodules showing active nodulation after 22 days of inoculation with the different strains. [file Image_2.TIF]

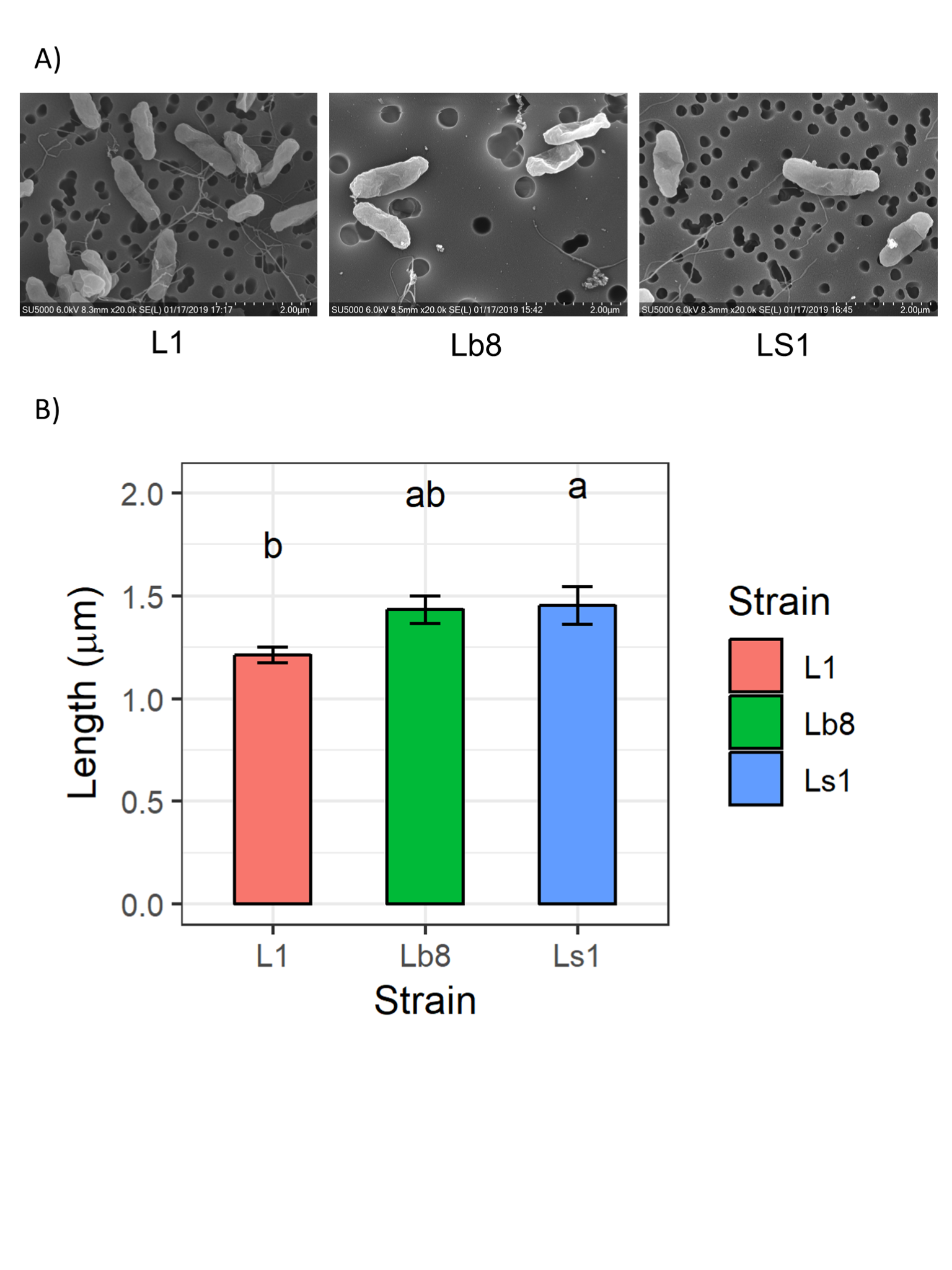

Supplement: Supplementary Figure 3 — (A) A scanning electron micrograph of the three strains L1, Lb8, and Ls1. (B) Bar plots showing the length of the different strains (n = 44). Same letters on top of each bar plot are not significantly different (HSD, p > 0.05). [file Image_3.TIF]

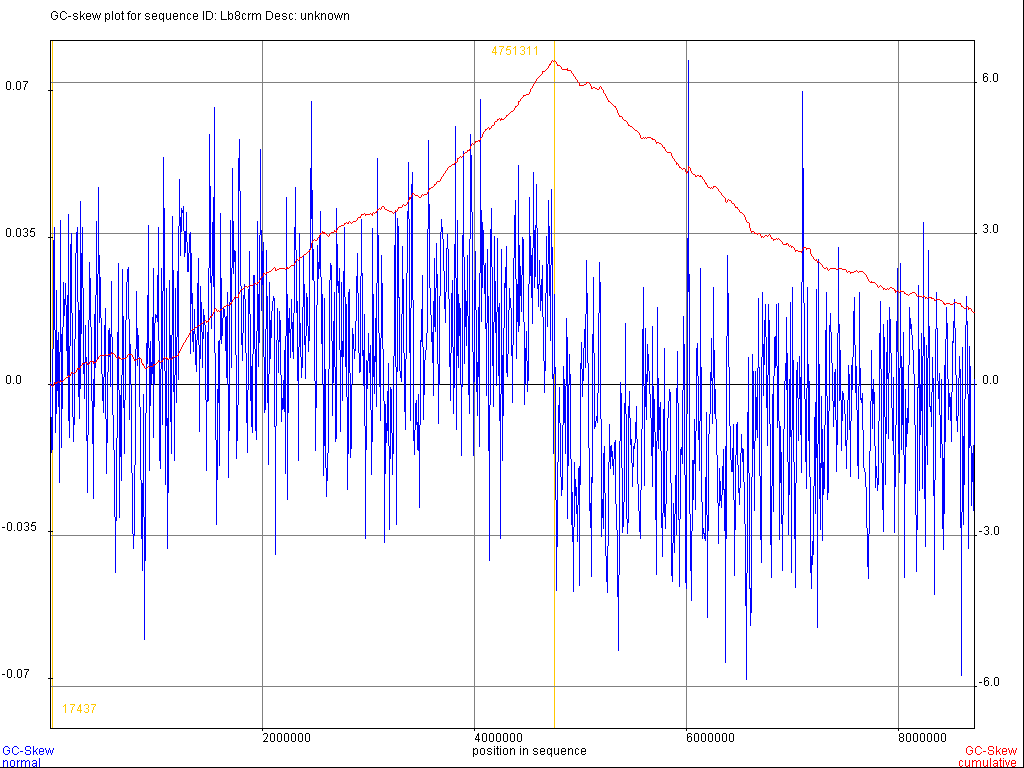

Supplement: Supplementary Figure 4 — A GC-skew plot showing the putative locations of the oriC and terminus in the Bradyrhizobium sp. Lb8 genome. [file Image_4.TIF]

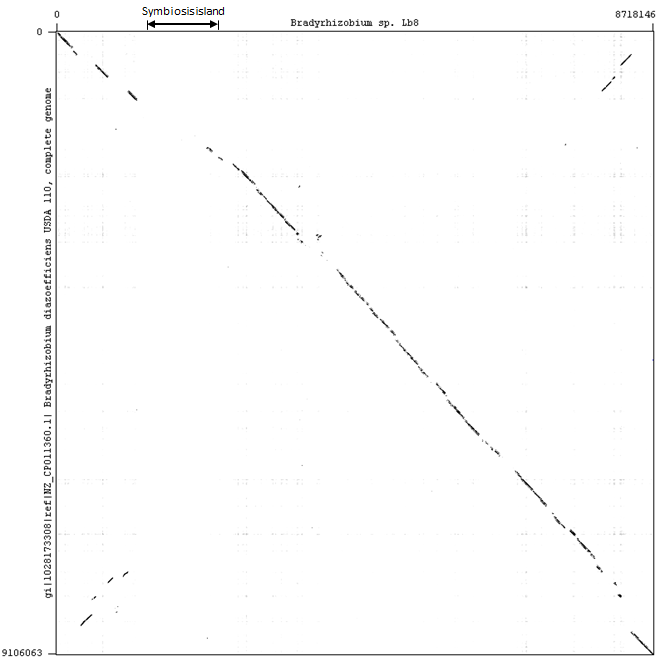

Supplement: Supplementary Figure 5 — A dot plot showing the similarity between Bradyrhizobium sp. Lb8 and B. diazoefficiens USDA 110T. [file Image_5.TIF]

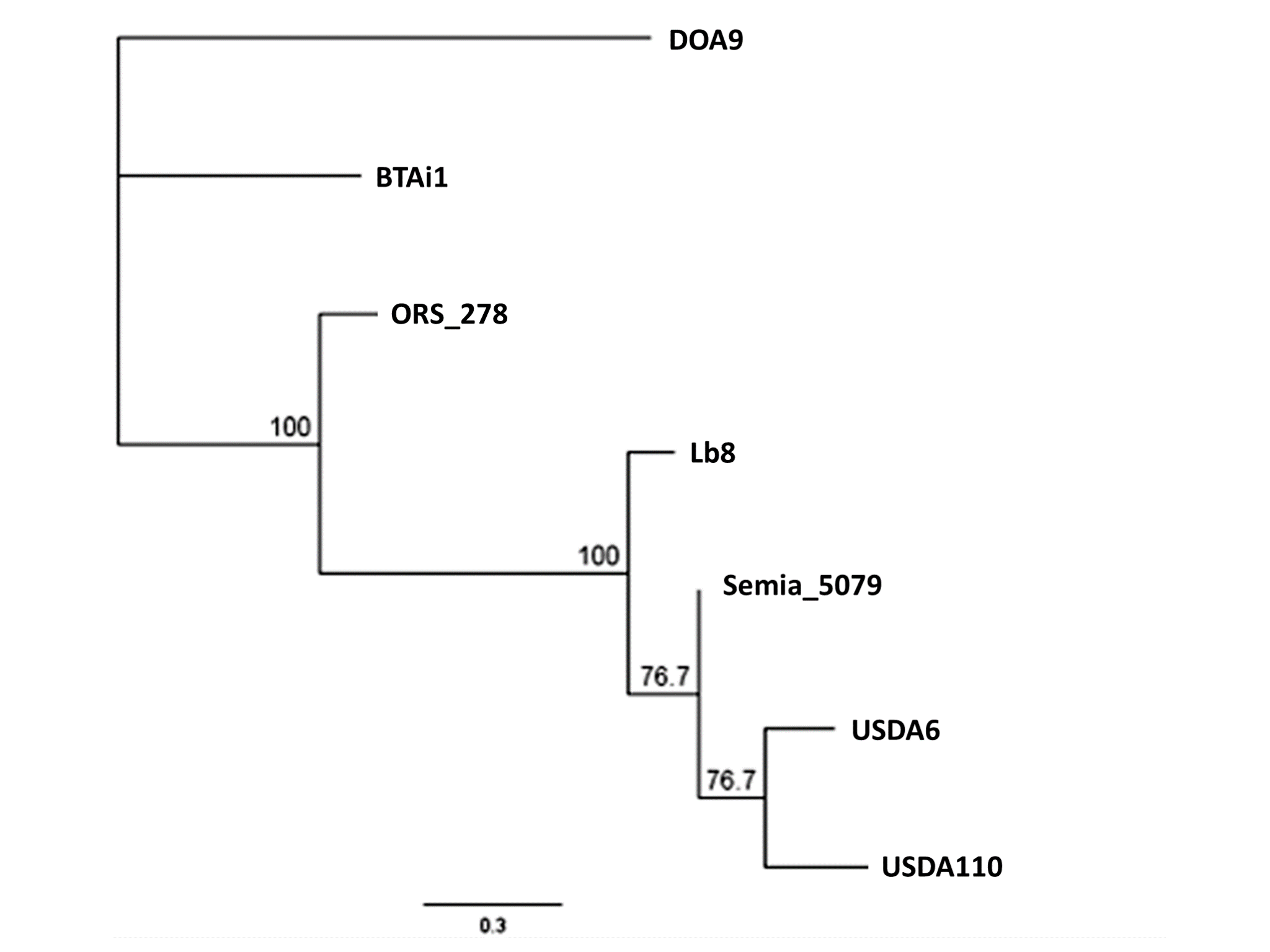

Supplement: Supplementary Figure 6 — Neighbor-joining tree based on concatenated sequences of nod genes present in Bradyrhizobium sp. Lb8 (nodU2DABCSU3I-effC-nodZNT) and closely related species within the genus Bradyrhizobium. The significance of each branch is indicated by a bootstrap value calculated for 1,000 subsets. Bar, 3 substitutions per 1,000 nucleotides. [file Image_6.TIF]

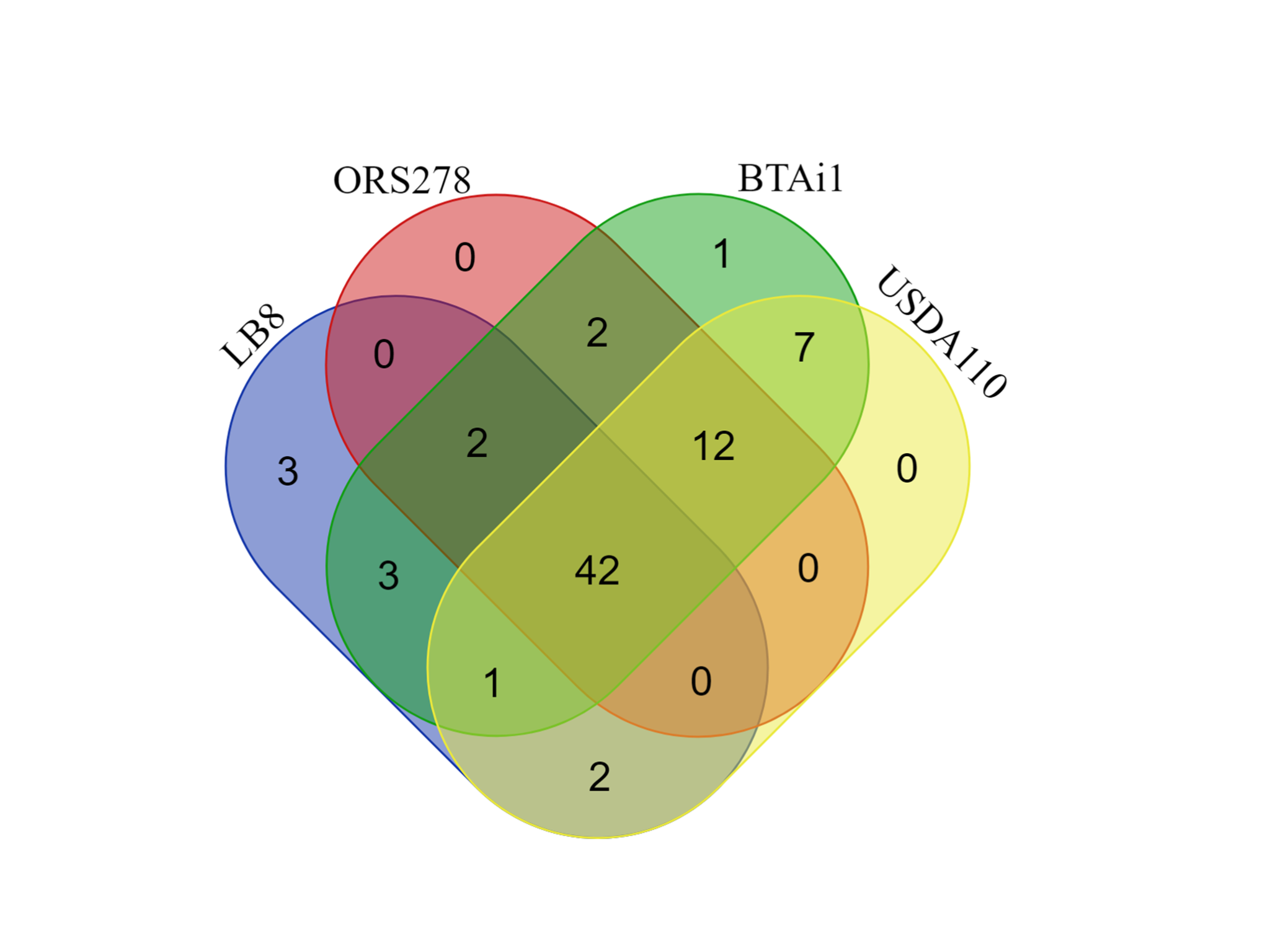

Supplement: Supplementary Figure 7 — The number of nitrogen metabolism-related genes that are present in the different Bradyrhizobium genomes. [file Image_7.TIF]
